# From preventive eugenics to slippery eugenics: Population control and contemporary sterilisations targeted to indigenous peoples in Mexico

**DOI:** 10.1111/1467-9566.13556

**Published:** 2022-10-04

**Authors:** R. Sanchez‐Rivera

**Affiliations:** ^1^ Gonville & Caius College Cambridge UK; ^2^ Department of Sociology University of Cambridge Cambridge UK

**Keywords:** eugenics, indigenous, Mexico, racism, reproductive justice, sterilisation

## Abstract

Eugenic ideas in Mexico were popularised after the Mexican Revolution (1910–1920) as a way of ‘modernising’ and ‘civilising’ the nation. As a result, eugenic ideas were able to linger and be maintained through different departments, institutions, and individuals from all disciplines. After eugenics was considered a pseudoscience, its practices and ideas continued through population control measures that targeted indigenous populations for sterilisation, a trend that still prevails. The purpose of this article is to explore the legacies of eugenics in current sterilizations procedures mostly targeted at indigenous communities in Mexico. I offer the term ‘slippery eugenics’ to account for the legacies of eugenics in Mexico which, in this specific case, resurface in the systematic forced and coerced sterilisation procedures targeted at indigenous communities.

Following the Mexican Revolution (1910–1920), eugenic ideas gained credibility in Mexico, specifically amongst the country’s elite. Eugenic policies were favoured as a method of keeping up with the ‘west’, reconstructing the nation after the revolution and ‘modernising’ and ‘civilising’ Mexico. Eugenic theories were also proposed to bolster the ideal of homogenisation, utilising mestizaje (mixedracedness) as the organising trope of the new revolutionary nation (Sanchez Rivera, 2019, p. 1). As Philippa Levine (2017, pp. 8–9) states, this trend was not uncommon in newly independent nations. In fact, ‘creating a biologically sound nation appealed alike to scientists and doctors looking to improve health and to politicians keen to consolidate their power’ (Levine, 2017, p. 9). As a result, eugenic ideas flourished across different institutions and individuals (Stern, 2011, p. 433). This article explores the legacies of eugenics in Mexico, specifically in relation to the systematic sterilisations of indigenous communities.

My hypothesis is derived from the analysis of archival materials obtained from the Centro Médico Nacional Siglo XXI‐Unidad de Congresos as well as legal documents and laws relating to family planning programmes in Mexico and on the analysis of works by groups and scholars who both critiqued and advocated population control policies. Documents relating to the case under analysis—which pertains to the sterilisation of a group of men in Guerrero—were found in the Archivo Histórico de la Comisión Nacional de los Derechos Humanos in Mexico City. This article contributes to scholarships in the fields of historical sociology and the sociology of health and illness as well as to a scholarship on racialisation and reproductive justice and marks an important development in the field of critical eugenics and its legacies. It raises several important questions, such as what is the link between eugenics and population control. As the title of this article alludes, I propose the concept of slippery eugenics, an original and innovative framework, for understanding the legacies of eugenics in Mexico.

## INTERROGATING POPULATION CONTROL MEASURES AND REPRODUCTIVE RESPONSIBILITY

Throughout the history of population control, individual and systematic pressure has been applied to ciswomen to regulate their reproductive capacity through a diverse array of technologies. By contrast, men have been largely excluded from or have not been expected to participate in ‘most governmental and non‐governmental initiatives with respect to reproductive health in general and birth control in particular’ (Gutmann, 2007, p. 108). At the beginning of population control measures in Mexico, new family planning programmes launched slogans like ‘responsible parenthood’ (Gutmann, 2007, p. 114) but placed reproductive responsibility solely on ciswomen. It was not until the international conferences in Cairo (1994) and Beijing (1995) that men were formally addressed, when doctors and nurses were trained to perform the no‐scalpel vasectomy (Gutmann, 2007, p. 111). Nonetheless, these practices were limited as men were seen as untrustworthy or as a threat to reproductive resources (Ibid).^1^ While the majority of the cases of forced and coerced sterilisation practices as well as family planning efforts were more generally directed to women, it is important to analyse how population control measures differently affect bodies along various axes of oppression like ‘race’, class and the legacies of colonialism (Hartmann, 2016; Ross, 2017).

Considering the accounts of forced and coerced sterilisation among indigenous populations in Mexico, the National Commission for Human Rights produced recommendations about the reproductive health of indigenous peoples in November 2001 (Meléndez, 2009, p. 159). General recommendation 4 denounced forced and coerced sterilisations as a violation of consent by the Health Secretariat (SSA) (Meléndez, 2009, p. 159). As a result, the Mexican government with the help of the SSA modified the Official Mexican Norm to ensure that no more permanent sterilisations occur. However, focussing on policy and ‘choice alone effectively privatises it: in making things a matter only for the individuals concerned [while] it occludes the wider social consequences’ (Madhok et al., 2013, p. 11). For instance, people in rural parts of Mexico resort to sterilisations or other forms of population control and support their rationale using neo‐Malthusian logics (Hirsch, 2008) that are not unsimilar to the rhetoric employed by the Mexican government after the 1970s.

The case I discuss appears among the files of the Commission for the Defence of Human Rights in the State of Guerrero (CDDHG) as Recommendation 35 of 2004 (CDDHG, 2004). This recommendation was made after various indigenous organisations denounced the International Labour Organization (OTI), the Education, Health, and Nutrition Program (PROGRESA) and the Direct Support Program of Rural Areas (PROCAMPO) for sterilising mostly indigenous people in exchange for money and services or for offering ‘false vitamins’ (i.e., contraceptives) during their family planning programmes (Meléndez, 2009, p. 159). In response, the human rights commission of Guerrero recommended the state’s Secretary of Health institute an administrative procedure for personnel from the sanitary jurisdiction of the State’s Services of Health in Costa Chica, who allegedly participated in the forceful sterilisation of men there. The Commission called for the application of sanctions by the Secretary of Health and demanded compensations for the victims who were not informed and who did not give consent for the procedure, as understood by the Mexican Norm (NOM‐005‐SSA2‐1993) of Family Planning Services. The Secretary of Health in the State of Guerrero refused the recommendation. Before exploring the inner workings of Mexican public policy and responses to forced and coerced sterilisation, I document the details of this case.

On the 15th of April of 1998, the health brigade^2^ of the Secretary of Health in Guerrero arrived at the community of El Camalote in the municipality of Ayutla de los Libres. The health brigade offered indigenous men with more than four children a vasectomy as a contraceptive method (CNDHM, 2007, p. 1). However, in the case report by the National Commission for Human Rights in Mexico, these indigenous men recount that they were coerced and that the health brigade asked them to get a vasectomy in exchange for a community clinic (CNDHM, 2007, p. 3). According to these testimonies, the brigade personnel told the men that they would have a clinic, a permanent medical doctor stationed in their community and access to necessary medicines if they underwent the procedure. The men also recounted being told that for those who accepted the above‐mentioned contraceptive methods, compensation would be offered in the form of food, clothes and child support. The men further allege that they were informed by the nurse from the brigade that their wives would be removed from the Education, Health, Nutrition Program (PROGRESA) if they refused the procedure. As a result, 13 of these men accepted the surgery, which was performed on the 16th and 17th of April 1998 (CNDHM, 2007, p. 1). Additionally, in 2001, another man made a formal complaint after he was subjected to a vasectomy with no scalpel in the Hospital Básico Comunitario of Ayutla de los Libres in Guerrero (CNDHM, 2007, p. 1).

In November 2003, the case was brought to the Commission for Human Rights in Guerrero by 13 indigenous men, claiming they had suffered human rights violations. As Madhok et al. (2013) explains, ‘the understanding of agency under coercive conditions remain undertheorized’ especially in settings and circumstances where deep‐rooted social inequalities prevail (Madhok et al., 2013, p. 7). In the case of population control practices which develop into coerced sterilisations such as this one, it is important to reflect on the role of social inequalities in this context. Historically, indigenous men in Mexico have been pathologised as ‘unfit’ to reproduce the nation. Their reproduction—as reflected in this case—was conceptualised as incompatible with the project of ‘mestizaje’ (which favoured reproduction between white men and indigenous women). Coercion can be identified here then as the ways in which the men were threatened to have important community resources removed if they refused the procedures. More implicitly, the historical norms which devalue and pathologise their reproduction also played a role. Thus, whilst they may tentatively have ‘agreed’ to the vasectomies, this ‘choice’ was shaped by various structural and cultural factors. In this case, the ‘associations between agency and coercion highlight, by contrast, the varying and unequal constraints within which we exercise our agency’ (Madhok et al., 2013, p. 8).

After the commission for human rights in Guerrero disregarded the 2004 recommendations, the UN also condemned these practices. In 2006, after revising the work done by the National Commission for Human Rights, the Committee on the Elimination of Racial Discrimination confirmed that systemic sterilisation practices targeting indigenous peoples in Mexico did occur (Meléndez, 2009, p. 159). According to Meléndez (2009), these accusations were denied by the Mexican government and its corresponding institutions, which include the Secretariat of External Relations, the Opportunity Program, Procampo Program and the National Commission for the Development of Indigenous Peoples (Meléndez, 2009, p. 159). The health sector did, however, admit the responsibility of individual personnel for the vasectomies in Guerrero (Meléndez, 2009, p. 159). This displacement of responsibility from institutions to individuals goes to the core of what I describe with my concept of ‘slippery eugenics’: the obscuring of structural dynamics in the form of population control, racism and other coercive policies targeting indigenous populations.

### Sterilisation and slow death in Mexico

In 2007, the National Commission for Human Rights of Mexico took up the case under the name of Recommendation 66l, resulting in a thorough investigation and a series of recommendations. The National Commission for Human Rights in Mexico undertook the case because, as they stated, the government of Guerrero failed to abide by local and international rules and norms and to respect the reproductive rights of indigenous populations. The National Commission that advocates for Human Rights in Mexico was however constrained, in so far as it can only make recommendations on the cases brought forward. It is the remit of the Secretary of Health, by contrast, to accept or decline such recommendations. The recommendations of the National Commission in this case were as follows:

(1) That the state’s Secretary of Health act against the people involved in the forced sterilisation of these men.
(2) Compensation is needed.
(3) Personnel need to be instructed further on how family planning services should be provided (CNDHM, 2007, p. 4)

Additionally, one of the rulings issued by the National Commission for Human Rights in Mexico is that the Health Brigade of Guerrero did not provide proper reproductive advice, specifically as they failed to inform the men of other contraceptive methods that were available. As a result, the commission called for a compensation of $300,000.00 M.N. each for the men involved. The National Commission for Human Rights concluded their report by recommending the Secretary of Health to accept the recommendation made by the commission of the state of Guerrero in 2004, within a period of 15 working days, or provide the evidence needed for their defence.

In response to the charges, the Secretary of Health denied having offered anything to the victims in exchange for sterilisation. However, the State Health Services failed to present any record of the mandatory counselling sessions required to ensure that the patients were informed of their reproductive rights and the contraceptive methods that were available. Here, the lack of counselling indicates that, most probably, the men had received little information about the procedure. Moreover, the consent forms they signed were not written in their mother tongue—Tlapaneca—and the men did not have access to an interpreter. This language barrier shows how indigenous peoples are seen as a homogeneous entity whose languages and culture are disregarded in favour of Spanish as the “main” mestizo language.

The Secretary of Health also stated that the men were never threatened with having benefits removed if they did not sign the consent form. I argue that the nature of ‘coercion’ in this case does not hinge on the issue of the usage of sterilisation as a contraceptive procedure but on the issue of consent. The framework of ‘slippery eugenics’ allows us to conceptualise the use of ‘consent’ and signed consent forms as a way to escape the state apparatus, discourses and practices surrounding human rights while continuing to employ systemic eugenic tools against a historically pathologised community. Borrowing from Jasbir Puar’s work, I argue that consent forms operate a ‘mode of neoliberal and affective capacitation or debilitation as mediated by different technological assemblages’ (Puar, 2017, p. 2). Puar’s usage of the framework of ‘slow death’ as the ‘debilitating ongoingness of structural inequalities and suffering’ is particularly illuminating when applied to this case (Puar, 2017, p. 1).

Puar borrows the concept of slow death from Lauren Berlant’s (2007) who devised the concept to explore how sovereignty and agency are constructed in relation to obesity. Berlant defines slow death as the ‘physical wearing out of a population and the deterioration of people in that population that is very nearly a defining condition of their experience and historical existence’ (Berlant, 2007, p. 754). Due to the colonial dynamics that constructed indigenous populations in Mexico as less than human, population control and forced sterilisation are other symptoms of the contemporary experience of historically exclusionary practices. Borrowing from Berlant, I argue that these practices have *slipped* into or become an ordinary part of a ‘normative form of governmentality’ (Berlant, 2007, p. 755) which targets indigenous groups. In this sense, indigenous populations in Mexico encounter ‘slow death’ through the debilitating ‘ongoingness’ of structural inequalities supported by the development and application of technologies (i.e., sterilisation) that were implemented to structure and produce the mestizo (Wade, 2017). Thus, in Mexico, ‘[b]iopower operates when a hegemonic bloc organizes the reproduction of life in ways that allow political crises to be cast as conditions of specific bodies and their competence at maintaining health or other conditions of social belonging; thus, this bloc gets to judge the problematic body’s subjects, whose agency is deemed to be fundamentally destructive’ (Berlant, 2007, p. 765). By structuring population control measures that systematically target indigenous people, different levels of Mexican society—from medical practitioners to the government—as well as external factors implement slippery eugenic practices, creating the ‘slow death’ of the country’s indigenous groups.

It is therefore necessary not only to implement local, national and international policies that protect and advocate for informed consent and which condemn forced and coerced sterilisation processes, but which question the structural dynamics of oppression and racism more widely. Such measures would problematise the status of indigenous populations in Mexico and their relationship with public health services in relation to different racialising assemblages (Weheliye, 2014) that construct the mestizo, the only body worth protecting. Similarly, the framework of slippery eugenics allows me to explore the different layers of complexity in relation to the legacies of eugenics in Mexico. Specifically, it brings our attention to the myriad actors involved; from individual acts of self‐regulation to governmental and non‐governmental agencies that advocate for the continuations of contemporary eugenic practices.

## FROM PREVENTIVE EUGENICS TO SLIPPERY EUGENICS

Eugenic ideas successfully pervaded Mexico’s social and institutional landscape during the post‐revolutionary period (1920–1950). Various societies, departments, laws and institutions advocated for eugenics from the beginning of the 20th century. These include the Department of Anthropometrics (1908), Public Health Department (1914), Department of Public Health‐School of Hygiene (1921), Department of Psychopedagogy and Hygiene (1925), the implementation of medical prenuptial certificates (Sanitary Code of 1926 and the General Population Law, 1928), School of Puericulture (1929), General Population Law (1936) and the Department of Racial Hygiene (1941) among many others (Horacio Reggiani, 2019; Stepan, 1991; Stern, 1999; Stern, 2011; Stern, 2016; Suarez y López, 2005; Turda & Gillette, 2014). These institutions, practices, laws and ideas are all connected to the emergence of eugenics and the creation of societies that advocated for eugenics as a tool to ‘better’ the Mexican ‘race’ during the first half of the twentieth century. These ideas did not disappear but continued to permeate throughout the second half of the twentieth century ‘as eugenicists continued to promote maternal assistance, the identification and rehabilitation of juvenile delinquents, and the incorporation of theories of heredity into the administration of health, education, and prisons’ (Stern, 1999, p. 387).

Nancy Stepan’s *The Hour of* Eugenics (1991) traces the development of eugenics in Mexico, Brazil and Argentina. Taking a social constructivist approach, Stepan (1991) demonstrates that different versions of eugenics were practiced in different contexts which, in her view, does not constitute a bastardisation of ‘Western’ science but the creation of other types of context‐specific sciences. In this sense, ‘the intellectual and scientific bedrock laid down in previous decades by both European scientific influences and what one scholar has called “creole science” set the stage for the extension of eugenics into the arenas of medicine, public health, law, and the social sciences’ (Stern, 2016, p. 2). Nonetheless, the type of science created in some contexts of Latin America was used transnationally and/or to understand other countries inside of the region (Hooker, 2017). Thus, Stepan created the basis for other works that legitimised the intellectual mechanisms and tools created by Latin American scientists, physicians and eugenicists. This is not to say that Latin American scientific production was homogeneous; on the contrary, there were many internal and external disputes. For instance, Stern (1999) identifies that there were disagreements between feminist eugenicists and male puericultors as the latter dominated the field of both eugenics and puericulture in Mexico (Stern, 1999, p. 375).

Stepan’s major contribution is the coining of ‘preventive eugenics’ (1991) to explain and describe Latin American eugenics. She argues that the outcome of ‘preventive eugenics’ was ‘directed to improving the nation by cleansing from the milieu those factors considered to be damaging to people’s hereditary health’ (Stepan, 1991, p. 17). According to Stepan, this type of eugenics did ‘less to improve public health in Latin America (most of the eugenicists’ social‐welfare recommendations were never implemented) than to promote new, biologically governed norms of social behaviour which were justified in the name of hereditarian science ‐ something new, modern, and in keeping with the scientific standards of Europe’ (Stepan, 1991, p. 17). Until the 1940s, Mexican eugenicists were more interested in Lamarckian principles of eugenics as a mode of endorsing their ‘racial project’ of mestizaje. By adopting and adapting Lamarckian ideas, eugenicists advocated for puericulture, pronatalism, conscious maternity and the creation and centrality of the ‘*gran familia Mexicana*’ (Sanchez‐Rivera, 2021; Stern, 1999; Turda & Gillette, 2014). After the 1940s, eugenicists in Mexico started to transition from Lamarckian eugenics to biotypology^3^ (Eraso, 2007) as a way of legitimising themselves and fighting against eugenics ‘fall from grace’—after the atrocities of the final solution—and the legitimation of Weismannian ideas over Lamarckian thought.^4^ Thus, racial thinking and eugenics are linked when it comes to Mexico as a “mestizo nation” (Moreno Figueroa & Saldívar Tanaka, 2015).

Mestizo literally translates to mixed race (Goldberg, 2009; Stolke, 2009, p. 2). This was one of the many categories that existed during the caste system in Latin America. At the end of the 19th century and beginning of the 20th century, mestizaje became a way of homogenising the population in Mexico (López Beltrán 2013, p. 391)—as almost synonymous to mexicanidad (Silva & Saldivar, 2018, p. 433). Mestizaje became a political and social ideology defined during the Mexican nation‐building process. In this process of making the mestizo the nation’s archetype (Wade, 2017), tensions regarding exclusionary practices and ideas around sameness and differences emerged (Wade, 2005, p. 240). Scholars present how the mestizo‐identifying subject can relate to ideas of ‘racial difference’ whilst simultaneously shifting to identify with “racelessness” by assuming, supporting and privileging the foundational myth of the mestizo (Moreno Figueroa, 2013; Wade, 2010; Wade, 2017, p. 51). This means that everything outside of the acceptable mixture created through this myth becomes ‘othered’.

Eugenics and mestizaje have a symbiotic relationship. During the Porfiriato (1876–1880; 1884–1911), Porfirio Díaz (1830–1915) and his board of intellectuals (*los científicos*) privileged a more Europeanised society and, with it, whiteness. Amid the revolution, however, Andrés Molina Enríquez (1868–1940) published Mexico’s *Big National* Problems (1909) in which he favoured mixedracedness as the only way for Mexico to fortify its modernity. Enríquez’s ideas became popularised by José Vasconcelos (1882–1959) in *The Cosmic* Race (1925). In part one of this book, Vasconcelos argues that there were three main stages in the creation and maintenance of the cosmic race. He named these periods the three laws of social states. The first stage is ‘the material or war‐based’ (Vasconcelos, 1925, p. 33). This was dictated by the Spanish colonisation and the forceful mixture as an outcome of sexual abuse. The second stage was ‘the intellectual or political’ characterised by the use of reason, law and science to manage the population—with eugenics as an example. Vasconcelos saw these laws and scientific practices as an imposition from the ‘West’ as ‘we [were] all prisoners of [their laws and science] and [in his view] it [was] important to get out of this stage’ (Vasconcelos, 1925, p. 34). Vasconcelos’ final stage was the ‘spiritual or aesthetic’. Here, reason and law are not the main driving forces to maintain the cosmic race but the ‘*aesthetic pathos’* (Vasconcelos, 1925, p. 34). To him, mixture would be dictated by love which would be guided by ideas of beauty and aesthetics (Vasconcelos, 1925, pp. 35–36). Nonetheless, his own ideas of beauty are racialised as he continuously invisibilises and disparages Black and Asian populations (Chang, 2017; Gleizer, 2015; Sanchez‐Rivera, 2020; Yankelevich, 2017).^5^

In her article titled ‘The Hour of Eugenics in Veracruz, Mexico’ (2011), Stern expands on the interconnectedness of racialising practices and ‘preventive eugenics’; using the eugenic law 121 of 1932 in Veracruz to achieve a broader understanding of the relevance of Stepan’s ‘preventive eugenics’. This allows Stern to conceptualise eugenics in other regions outside mainstream scholarly works that are usually constricted to the United States and Europe (Stern, 2011, p. 431). Stern argues that the law implemented in Veracruz did not come in a vacuum. Conversely, Mexico’s post‐revolutionary ideas and eagerness to modernise made it ‘fertile ground’ for the acceptance of these kinds of eugenic measures which made possible the creation, cementation and maintenance of ‘the cosmic race’. According to Stern (2011), the socialist governor of Veracruz Adalberto Tejeda had a personal library including books that advocated for the superiority of racially mixed types and the veneration of the ‘cosmic race’ (i.e., the mestizo) (Stern, 2011, p. 439).

By exploring the case of Mexico—where eugenic programmes were still active during the 1960s—Stepan and Stern demonstrate that eugenic programmes and ideas did not end when ‘the world began to gain awareness of the horrific consequences of Nazi Germany’s racial hygiene programme’ (Stern, 2011, p. 433). The case of Veracruz demonstrates how preventive eugenics gained ground in Mexico only to show one of its most extreme manifestations in the first half of the 20th century (Stern, 2011, p. 442). In this sense, Stern’s work both challenges and confirms the existence of Stepan’s preventive eugenics. One of Stern’s most thought‐provoking claims is that many of the legacies of eugenics are ‘palpable today even as its signature features of racial bias and reproductive control often are viewed as undemocratic and antithetical to human rights’ (Stern, 2016, p. 2).

Using my framework of ‘slippery eugenics’, we can conceptualise how eugenic ideas and practices remain pervasive in contemporary Mexico. In a manner, slippery eugenics echoes Stepan’s conception of ‘preventive eugenics’ as it shakes the periodisation of eugenics in Mexico. It adds further to this conceptualisation by focussing on how the legacies of eugenics manifest today through state policies and also through individual self‐regulation via internalised eugenic practices and ideas. In her work on mestiza subjectivities, Moreno Figueroa (2006) coins the term ‘slippery emotion’ to show how ‘expressions of racism have infiltrated social life and transformed their modes of presentation’ (Moreno Figueroa, 2006, p. 17). Considering the long history of eugenics in Mexico, ‘slippery eugenics’ unveils how practices and ideas have infiltrated structural dynamics and the everyday life of individuals to make contemporary forms of eugenics imperceptible. Moreover, ‘slippery eugenics’ provides the tools to understand how the legacies of ‘race’ science have penetrated social, scientific, medical and cultural understandings of Mexican nationalism as a ‘mestizo nation’.

I coined the term slippery eugenics as a way of integrating the concepts of preventive eugenics (Stepan, 1991), laissez‐faire eugenics (Sleebom‐Faulkner, 2011) and flexible eugenics (Taussig et al., 2005). Preventive eugenics (1991)—as discussed above—shows how states and influential actors implemented biologically governed norms of social behaviour which were justified in the name of hereditarian science (17). I argue that the legacies of these norms remain in part, but that they have also taken different shapes in line with neoliberal governance. In Sleebom Faulkner’s article on the practice of genetic testing in China, she explains how many couples might choose to abort abnormal foetuses which denies the applicability of state eugenics (2011, pp. 1802, 1809). Nonetheless, she explains how this ‘individual choice is moulded by a constellation of pressures’ as ‘a lack of financial support and welfare institutions, as in many developing countries, limits the couples’ ability of raising a child with a birth defect and looking after ‘handicapped’ grown‐ups (Sleebom‐Faulkner, 2011, p. 1809). Applying laissez‐faire eugenics, I account for these ‘choices’ by analysing the structural inequalities that shape individual decision‐making processes. Additionally, laissez‐faire eugenics occurs when the state does not or is not expected to control the population through eugenics. Today, eugenics is so engrained in the everyday lives of individuals that the state and institutions can take a laissez‐faire approach to these measures, which manifests instead as ‘common sense’ choices. Finally, flexible eugenics describes when bodies historically constructed as atypical or abnormal meet the possibility of technological self‐regulation or betterment. This term is used by Taussig et al. (2005) to refer to the tension that ‘little people’ encounter when presented with discourses of individual perfectibility and a collection of what they term as ‘technically mediated choice’ (Taussig et al., 2005, pp. 195–196). In this sense, groups that have been historically pathologised or categorised as ‘non‐human’ or ‘less‐than‐human’ internalise eugenic measures and self‐regulate accordingly. I argue that the division of these forms of eugenics in Mexico are often blurred, and a mixture between state intervention, individual self‐regulation and the mediation of other institutions that are not particularly constricted to the state is visible. I coin the term ‘slippery eugenics’ to account for and illustrate this melange of practices and ideas.

In Mexico, contemporary eugenic practices tend to follow a combination of preventive, flexible and laissez‐faire eugenics which operate in tandem and collaboration with each other. Through the practices of forced and coerced sterilisations targeted to indigenous communities in Mexico, I observe how eugenics never stopped. Rather, eugenic ideas and practices merely slipped through the cracks of governmental policies and individual understandings of belonging that are then translated to coercive practices by the medical personnel pressured by the long history of pathologisation of indigenous communities. Overall, slippery eugenics accounts for the ongoing development and impact of eugenic ideas in Mexico, which continue to shape the reproduction of the nation into the present.

## POPULATION CONTROL AND THE SURGE OF SLIPPERY EUGENICS AFTER THE SECOND HALF OF THE 20TH CENTURY

The permanent secretary of the Mexican Society of Eugenics, Alfredo Saavedra, continued writing about eugenics until the mid‐1970s (Stern, 2011, p. 433). Similarly, Mexican institutions that previously advocated for eugenic ideas, pronatalism and puericulture changed their name and continued operating under similar bases. For example, a decade after its foundation in 1929, the Mexican Society of Puericulture was renamed as the Mexican Society of Paediatrics (Frenk & Avila‐Cisneros, 1991, p. 68). In this Section I, document how different population control measures developed after the second half of the 20th century, to demonstrate how eugenics slipped into contemporary institutions and practices. Using documents written during the second half of the 20th century that either advocated or critiqued population control in Mexico, I devise a comprehensive analysis of population control policies and ideology during this period.

Before exploring the Mexican context, it is imperative to survey the surge of rhetoric around population control globally as this had a major impact in later policies regarding family planning in the country. Conelly (2008) argues that the demographic revolution or the global growth of the population between 1890 and 1940 shakes the periodisation of the 20th century (4). Conelly (2008) states that ‘nativism, eugenics, pronatalism, and coercive or manipulative forms of family planning share a common history’ as ‘these ambitious population control schemes […] aimed to remake humanity by controlling the population of the world, typically by reducing the fertility of poor people and poor countries’ (xii). These discourses have racialising elements as in the beginning of the 20th century there were concerns about ‘racial suicide’ (when promoting pronatalism for Whites) but once the life expectancy of the ‘global south’ started growing in the 1940s–1950s ‘population growth began to appear as a global crisis’ (Conelly, 2008, pp. 7–8; Ross, 2017, pp. 18, 30–41).

Population control is based on Thomas Robert Malthus’ (1766–1834) *Essay on the Principle of Population* (1798) which argued that there were insufficient global resources to support an exponentially expanding population. An inspiration for Darwin (1809–1882) and Galton (1822–1911), Malthus’ ideas created widespread preoccupation that ‘it would be the poorest and most fecunt examples of humanity who would overrun all the rest’ (Conelly, 2008, p. 2).^6^ After eugenics fell from acceptability, population control gave elites the tools to implement policies regulating reproduction all over the world. This allowed governmental organs and private funders to scrutinise the reproductive capacity of fertile people to ‘pursue goals associated with power, wealth, status, and property, creating difficulties and particular degradations for fertile and reproducing persons because of their sex and gender and their capacity to give birth to new life’ (Ross & Solinger, 2017, p. 13). Slippery eugenics accounts for the mixture of governmental and non‐governmental sectors that joins forces to control and manage the population through reproductive technologies and measures; and Mexico was no exception.

During the early 1970s, child mortality decreased, allowing Mexico’s population to grow substantially (Figueroa Perea, 1994; Gutmann, 2007). When Luis Echevarría started his campaign for the presidency in 1970, ‘Mexico’s population was 48.3 million; by the end of his term in office, 6 years later, it had grown to more than 70 million’ (Soto Laveaga, 2009, p. 2107). Before 1973, in line with pronatalism, contraceptives in Mexico were restricted by the Mexican Health Code (Figueroa Perea & Aguilar Ganado, 2006; Gutmann, 2007; Rodriguez‐Barocio et al., 1980, p. 3). Due to the amendment of Article 4 of the constitution in the early 1970s, the government started offering contraceptives through governmental family planning services. This Article stated that ‘men and women are equal under the law [and that] every person has a right to decide freely, responsibly, and informed, the number and spacing of their children’ (Secretaría de Salud, 2015). Additionally, these new changes in policy coincided with the creation of the new General Population law of 1974 which was overseen by the National Population Council (CONAPO) (Ley General de Población, 1974; Pullum et al., 1985, p. 42). In a neo‐Malthusian tone, and prioritising economic over social considerations, this population law advocated for a demographic plan proposing smaller nuclear families.

These new laws marked a changeover regarding population control policies in Mexico, which coincided with international pressures (i.e., the UN’s demographic and economic plans presented in Bucharest (1974) and US‐affiliated agencies like the World Bank) (Hartmann, 2016, pp. 102–104; Gutmann, 2007, p. 115), the nationalisation of steroid hormone industry, the need for creating medicines domestically, the need for supporting the Mexican health‐care system and reducing international dependency (Soto Laveaga, 2009, p. 2305). Due to the systemic disregard for rural communities in Mexico, Echevarría employed diverse ‘populist’ methods to supposedly help the working class—which was, for the most part, indigenous (Rand Oakley & Rodriguez, 2005). Using terms like ‘poor’ and/or ‘working‐class’, Echevarría invisibilised the systemic racism created by mestizaje by making it seem like a mere class and a geographical issue. In this sense, the eugenic categorisation of the ‘races’ becomes invisibilised by a ‘class’ rhetoric that overlooks social inequalities produced by structural racism.^7^ Concurrently, the International Conference on Population and Development was celebrated in Bucharest in 1974 (Conelly, 2008, p. 299; Hartmann, 2016, p. 102). This conference focussed heavily ‘on the relationship between population issues and development’ (Conelly, 2008, p. 310; Hartmann, 2016, pp. 102–104; United Nations, 1974) which was used in Mexico as justification for their new population control plan.

Mexican policymakers started to change their approach from pronatalism, instead seeking ways of reducing fertility rates (Gutmann, 2007, pp. 112–114). This became evident through the new governmental rhetoric that framed population growth as a problem for modernity and progress. The administration of President José López‐Portillo (1976–1982) was determined to devise new family planning measures to continue with national and international trends of population control (Gallegos et al., 1977, p. 197). This new governmental programme was mostly targeted to the rural zones (Ibid), who were mostly indigenous. In this way, through family planning programmes, population control measures provided the means to continue the historical trend of racialising and pathologising indigenous communities.

In 1977, a new proposal for family planning was presented in Mexico to reduce fertility rates, which led to a new policy regarding these new demographic developments of population growth which was overseen by the coordinating office of the National Family Planning Programme (Rodriguez‐Barocio et al., 1980, p. 3) This new plan, according to Rodriguez‐Barocio et al. (1980), consisted of ‘a governmental body [that] was made responsible for getting family planning services to all couples who needed them, and for reducing [Mexico’s] growth rate to 2.5% annually by 1982’ (2). The National Plan for Family Planning in Mexico aimed to reduce fertility rates from a 2.5% to a 1% growth rate by the year 2000 (Gutmann, 2007; Nagel, 1979; Rodriguez‐Barocio et al., 1980, pp. 2–9).^8^

During the first six‐year term (1976–1982), the National Family Plan in Mexico secured its national goals for fertility reduction. There were two surveys in this period: The Mexican Fertility Survey (MFS) in 1976 and the Contraceptive Prevalence Survey (CPS). The MFS was the first fertility survey carried out in Mexico on a national level (Pullum et al., 1985, p. 40), and the latter was carried out to ‘establish a baseline data for the national planning programme and to determine what impact the programme had in its first 18 months of operation’ (Rodriguez‐Barocio et al., 1980) This led to a celebratory interpretation of the success of the programme once the survey findings were published in March 1979 (Lapham & Mauldin, 1985, pp. 122–123). For instance, Rodriguez‐Barocio et al. (1980) frame population growth as a problem and praise the data produced by the CPS, describing it as ‘the most far‐reaching innovative [family planning programme] in the non‐Communist world’ (Rodriguez‐Barocio et al., 1980, p. 4). According to Figueroa Perea and Aguilar Ganado (2006), the ‘success’ of these surveys obscured all the criticisms that arose from the practices of forced and coerced sterilisation by arguing that the population’s demands were being met.

In the early 1980s, fertility rates continued to decline at a moderated rate in comparison to the first six‐year term (Wulf, 1982, p. 136). By 1982, the National Population Council did a National Demographic Survey (NDS) that explored the goals made by the National Family Plan (Pullum et al., 1985, p. 40). This survey demonstrated that the greatest fertility decline was among ‘less‐educated’ and rural women, the prime targets of the government programme. Women in the highest level of schooling, for whom the total fertility rate was already about three children, showed virtually no change in their fertility (Pullum et al., 1985, p. 40). Reading literature of the time, it is notable that economic considerations and ideas around modernity and progress were being given more attention than the reproductive autonomy of the women being surveyed. For instance, proponents of population control measures argue that ‘further analysis [was] required for a clearer understanding of the relative importance of changes in preferred spacing of children, preferred family size and preferred ages at childbearing’ (Pullum et al., 1985, p. 45). Thus, these governmental programmes and surveys were based upon reproductive oppressions that ‘stem from a determination to exercise power over vulnerable persons and achieve goals that have nothing to do with the well‐being or interests of individual reproducers’ (Ross, 2017, p. 6).

Providing information regarding family planning programmes was not the main goal of the Mexican government. In a study carried out by Korzenny et al. (1983) to investigate the best mass media tools to spread information about family planning, they state that ‘government and private enterprises in Mexico launched “family planning entertainment programs”’ (238) like soap operas, posters and printed press. They also argue that the family planning programme in Mexico was ‘not geared towards teaching detailed information about the process or methods of contraception’ (Korzenny et al., 1983, p. 239). According to different scholars, the sterilisation practices done through family planning programmes were carried out in direct violation of Article 4 of the constitution, as people who underwent these procedures were not provided with adequate information, whilst others were given insufficient time between pregnancies, to name but a few of the contraventions (Figueroa Perea & Aguilar Ganado, 2006; Meléndez, 2009).

The second six‐year term of the National Family Plan was received differently by various groups and institutions—from the UN and the Mexican government to different feminist activist groups in Mexico (Gutmann, 2007). During the Second World Population Conference hosted in Mexico City in 1984 (Hartmann, 2016, p. 115), the United Nations (UN) ‘acknowledged the need to reward the Mexican government for its “successful” population policy’ (Conelly, 2008, p. 352; Figueroa Perea & Aguilar Ganado, 2006, p. 4). This was based on the population control measures carried out during the first six‐year term and disregarded the critiques made by various sectors of the population regarding practices of forced and coerced sterilisation. As a result, a group of researchers and activists created the National Survey on Risk Factors in Hormonal Contraceptives in the same year which concluded that forced and coerced sterilisation practices were being targeted mostly towards vulnerable groups (i.e., indigenous and rural communities) (Figueroa Perea & Aguilar Ganado, 2006, p. 5; Meléndez, 2009, p. 157). This resulted in the creation of different teaching guides by the government and researchers for medical personnel in 1986 (Figueroa Perea & Aguilar Ganado, 2006, p. 6). By 1987, the people interviewed in the 1984 survey were ‘followed‐up’. In this case, those administering the initial survey realised that the forced and coerced sterilisation procedures continued to rise. Since this report was published in 1988, it was not implemented for the next six‐year National Family Plan (1988–1994) (Figueroa Perea & Aguilar Ganado, 2006, p. 8).

In the 1990s, international and national groups and institutions critiqued demographic plans which, in turn, marked a changeover to policies and measures of population control. In 1993, the Mexican League for Human Rights stated—in their fifth conference of the child—that 528,000 Mexican women were sterilised without the complete information to make this decision (Meléndez, 2009, p. 159). Additionally, out of 2,300,000 people sterilised, 1,000,000 did not sign consent forms (Meléndez, 2009, p. 159). A year later, during the International Conference on Population and Development in Cairo (1994), demographic goal plans—like the one Mexico implemented in the 1970s—were condemned by different organisations that advocated for reproductive health. Similarly in Mexico, during the same year, the Ford Foundation and the UN Population Council Fund financed a new research project to explore reproductive policies in Mexico. This was cancelled by Mexico’s Health Secretariat, illustrating a clear disregard of and an attempt to invisibilise the sectors being systemically targeted for coerced or forced sterilisation (Figueroa Perea & Aguilar Ganado, 2006, p. 9). Similarly, national surveys in 1995 cited limited knowledge as the reason for not using contraceptives and Barber (2007) argues that while ‘information is available about whether family planning advice was received […] the comprehensiveness of the family planning advice was not assessed’ (10–11). It can thus be inferred that the Mexican government wanted to continue with their population control measures while giving little to no information about the process, despite the growing opposition from both local and international spheres.

Mexico is not an isolated case. Forced and coerced sterilisations and human rights violations occurred all over the region. Between 1996 and 2001, a sterilisation campaign targeted more than 300,000 indigenous women in Peru (Vasquez Del Aguila, 2022). Then, President Alberto Fujimori (1990–2000) appeared at a United Nations conference on women in Beijing (1995) where he defended family planning measures as a ‘tool to fight against poverty and social injustice’ (Vasquez del Aguila, 2006). Fujimori used the myth of population control tied with economic underdevelopment to further oppress a historically marginalised and racialised group. Similarly, in lieu of an official policy for sterilisation in Argentina and Brazil, the popularisation of biotypology and endocrinology ‘converged in some clinical settings to offer a scientific rationale for extralegal sterilizations of women who were labelled dysgenic and unlikely to produce robust children’ (Stern, 2016, p. 12). In Brazil, the 1997 family planning law had a goal to enable sterilisation in public hospitals (Amaral, 2019, p. 2). Here, cases of forced sterilisation, denial of services and requiring the partner’s consent for procedures denied fertile bodies the right to choose (Almeida & Silva, 2019; Edu, 2015, 2018).

## CONCLUDING REMARKS

The history of eugenics in Mexico has had a substantive impact on how we understand society today. In their advocacy of mestizaje, various different laws, institutions and academic works have successfully pervaded contemporary society. Here, I have discussed the legacies of eugenics after it ‘fell from grace’ through different population control programmes which targeted indigenous populations through sterilisation practices during the second half of the 20th century. These programmes facilitated the systemic sterilisation of indigenous communities on a national scale. Despite the creation of policies that protected ‘human rights’, it is important to question the racialisation processes behind what it means to be ‘human’ in the first place. Indigenous communities have been systematically degraded for centuries, which raises the question of how they can be integrated into the realm of human rights when there has been a systematic questioning of their humanity since the colonisation of New Spain.

These cases of reproductive (in)justices committed by the state against indigenous populations in Mexico constitute a clear example of the paradoxical fissure between policy and its implementation. The case of the indigenous men in Guerrero who were coerced into getting a vasectomy shows how ‘slippery eugenics’ operates in the Mexican context. First, the fact that the Commission for Human Rights in Mexico can only make recommendations to the Secretary of Health displays the inability of the system and the lack of regard of ‘human rights’. Additionally, the aspect of coercion must be further theorised to understand the dynamics of agency in a coercive setting. I argue that slippery eugenic is a useful term for understanding the legacies of eugenics in Mexico as it accounts for structural racism and social inequalities that come with the making of the nation through mestizaje. Through offering the novel concept of ‘slippery eugenics’, I provide the tools to continue researching eugenics as an ‘ongoing’ process. In short, the furthering of the conceptual knowledge of critical eugenic studies through slippery eugenics, highlights the combination of different forms, actors and legacies of eugenics. This, in turn, allows us to understand the legacies of eugenics in a multifaceted way that accounts for a more intersectional understanding of eugenic practices today.

## AUTHOR CONTRIBUTION

**Rachell Sanchez‐Rivera**: Methodology; Data curation; Writing – review & editing; Writing – original draft.

## Data Availability

The data used here was found in different archives in Mexico as well as secondary sources that both critique and advocate for population control.

## ENDNOTES

^1^
The *Oferta Sistemática* (literally translated to Systematic Offer) were ‘state mandated policies sought to increase the adoption and employment of birth control by women’ which meant that every time a cis woman would encounter a health professional for any reason, they would be offered contraceptives as a way of complying with quotas imposed (Gutmann, 2007, p. 110; Meléndez, 2009).

^2^
According to the General Secretary of Mexico, health brigades are constituted by medical personnel and/or nurses, engineers of industrial security, technicians of health and hygiene and social workers. These go to small communities and enterprises on specific dates to bring health services and consultancy.

^3^
“The scientific classification of bodies, endocrine therapy, and sterilization[…] Biotypology became a discipline devoted to providing instruments with which to gauge this integral approach to patients, with the aim of enabling physicians to diagnose and treat more effectively” (Eraso, 2007, p. 793, p.793)

^4^
By 1941 the Mexican state alongside Mexican eugenicists created the Department of Racial Hygiene; similarly, indigenist Manuel Gamio (1883–1960) supported ideas of biotypology in the American Scientific Congress of 1942 (Stern, 2000)

^5^
Indigenist Manuel Gamio makes claims supporting mixed‐racedness through his works which he then put into practice with the 1921 contest ‘*La India Bonita’*. This contest looked for the ‘most beautiful’ indigenous woman as a way of making them more attractive to White men (Ruiz, 2001; Samaniego, 2018; Samano Verdura, 2010; Sanchez‐Rivera, 2021; Zavala, 2006).

^6^
According to Hartmann (2016), population control relied upon the following suppositions: (1) Rapid population growth is a primary cause of the Third World’s development problems. (2) People must be persuaded—or forced, if necessary—to have fewer children without fundamentally improving the impoverished conditions in which they live. (3) Given the right combination of finance, personnel, technology and western management techniques, birth control services can be “delivered” to Third World women in a top‐down fashion and in the absence of basic health‐care systems. In both the development and promotion of contraceptives, efficacy in preventing pregnancy should take precedence over health and safety concerns. (23)

^7^
This also coincided with the discovery of the diverse purposes and benefits of the *barbasco* and the exploitation of this root and the—mostly indigenous—people whos cultivate it under dire conditions as a ‘strategy to building [Mexico’s] own pharmaceutical industry’ (Soto Laveaga, 2009, pp. 2300–2305).

^8^
This demographic plan was going to be divided according to Mexico’s presidential and political terms, hence, 6‐year terms. The first one being from 1976 to 1982; then 1982–1988; 1988–1994 and, lastly, 1994 to the year 2000 (Rodriguez‐Barocio et al., 1980, pp. 2–9; Nagel, 1979).
